# High‐resolution three‐dimensional chromatin profiling of the Chinese hamster ovary cell genome

**DOI:** 10.1002/bit.27607

**Published:** 2020-11-20

**Authors:** Stephen Bevan, Stefan Schoenfelder, Robert J. Young, Lin Zhang, Simon Andrews, Peter Fraser, Peter M. O'Callaghan

**Affiliations:** ^1^ Nuclear Dynamics Programme, The Babraham Institute Babraham Research Campus Cambridge UK; ^2^ Epigenetics Programme, The Babraham Institute Babraham Research Campus Cambridge UK; ^3^ R&D Cell Engineering, Lonza Biologics Little Chesterford UK; ^4^ Cell Line Development, World Wide Pharmaceutical Sciences, BioTherapeutics Research and Development Pfizer Inc. Andover Massachusetts USA; ^5^ Bioinformatics Facility The Babraham Institute Cambridge UK; ^6^ Department of Biological Science Florida State University Tallahassee Florida USA

**Keywords:** 3D genome organization, Chinese hamster ovary (CHO), genome assembly, Hi‐C, promoter interactome

## Abstract

Chinese hamster ovary (CHO) cell lines are the pillars of a multibillion‐dollar biopharmaceutical industry producing recombinant therapeutic proteins. The effects of local chromatin organization and epigenetic repression within these cell lines result in unpredictable and unstable transgene expression following random integration. Limited knowledge of the CHO genome and its higher order chromatin organization has thus far impeded functional genomics approaches required to tackle these issues. Here, we present an integrative three‐dimensional (3D) map of genome organization within the CHOK1SV® 10E9 cell line in conjunction with an improved, less fragmented CHOK1SV 10E9 genome assembly. Using our high‐resolution chromatin conformation datasets, we have assigned ≈90% of sequence to a chromosome‐scale genome assembly. Our genome‐wide 3D map identifies higher order chromatin structures such as topologically associated domains, incorporates our chromatin accessibility data to enhance the identification of active *cis*‐regulatory elements, and importantly links these *cis*‐regulatory elements to target promoters in a 3D promoter interactome. We demonstrate the power of our improved functional annotation by evaluating the 3D landscape of a transgene integration site and two phenotypically different cell lines. Our work opens up further novel genome engineering targets, has the potential to inform vital improvements for industrial biotherapeutic production, and represents a significant advancement for CHO cell line development.

## Graphical abstract

In this work Bevan and colleagues generate a comprehensive 3D genome map of a site‐specific integration (SSI) CHO host cell line using a combination of ATAC‐Seq, Hi‐C, and Promoter‐Capture Hi‐C. These datasets are used to identify higher order chromatin structures such as topologically‐associated domains (TADs), and puts them in the context of a genome‐wide 3D promoter interactome. The combination of such datasets may be used to design cell engineering approaches for improving CHO performance in the bioprocessing environment.

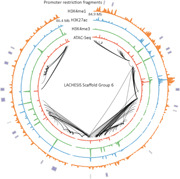

## 1 INTRODUCTION

The vast majority of recombinant therapeutic proteins are produced industrially using Chinese hamster ovary (CHO) cell lines. These cell lines have become the pillars of the biopharmaceutical industry, primarily due to their ability to produce large amounts of human‐compatible transgenic protein. Previous publications of iterative Chinese hamster and CHO cell line reference sequences (Lewis et al., 2013; Rupp et al., 2018; Xu et al., 2011) led to the production of a number of “omics” datasets, all aiding to increase our knowledge of recombinant CHO cell lines. Transcriptomic and epigenetic profiling has demonstrated the relationship between epigenetic modifications, changes in gene expression, and the phenotypic output of CHO cell lines (Feichtinger et al., 2016; Hernandez et al., 2019; O'Brien et al., 2018). These studies have also begun to establish the functional annotation of the CHO genome, including the identity of putative regulatory elements and noncoding transcripts (Feichtinger et al., 2016; Hernandez et al., 2019). Unfortunately, chiefly due to the fragmented nature of recent genome assemblies, a void of knowledge on the three‐dimensional (3D) genome organization within this cell type exists.

Our understanding of 3D genome organization and its importance in the regulation of many cellular processes has been revolutionized by the advent of proximity‐based ligation assays (Reviewed in Grob & Cavalli, 2018). These “C” techniques measure the contact frequency of loci either physically interacting or occupying close spatial proximity within the 3D chromatin architecture. This hierarchical architecture includes A/B compartments (Lieberman‐Aiden et al., 2009), topologically associated domains (TADs; Dixon et al., 2012; Nora et al., 2012), and chromatin loop structures (Rao et al., 2014). A/B compartments function to segregate transcriptionally active (“A”) and inactive (“B”) regions of the genome correlated by gene content, chromatin accessibility, and specific histone modifications (Lieberman‐Aiden et al., 2009). TADs are highly self‐interacting, submegabase regions of chromatin that possess significantly more intradomain interactions than interdomain interactions, and are enriched for CTCF at their boundaries (Dixon et al., 2012; Nora et al., 2012).

Interactions between an array of distal regulatory elements and target core promoters function spatiotemporally to modulate gene expression. These distal regulatory elements can reside large genomic distances away from their target promoters, circumvent more proximal promoters, and are proposed to exert their influence on transcription via chromatin loops (reviewed in Schoenfelder & Fraser, 2019). Higher order genomic segregation, into TADs for example, restricts the regulatory scope of these distal regulatory elements, which, if perturbed, has been shown to result in significant developmental defects (Lupiáñez et al., 2015; Symmons et al., 2016). Promoter Capture Hi‐C (PCHi‐C), a technique that enriches Hi‐C libraries for promoter contacts (Schoenfelder et al., 2015), has been used to link promoters to noncoding disease variants (Javierre et al., 2016), and uncover the importance of long‐range enhancer contacts in mouse embryonic stem cell pluripotency (Novo et al., 2018). Amongst others, these studies illustrate the importance of an increased understanding of 3D genome organization and its relationship to gene expression and ultimately cellular phenotype.

Despite the importance of CHO cells within the biopharmaceutical industry, the 3D chromatin architecture and in particular, how it affects the performance of recombinant cell lines has largely been unstudied. Current demand for complex biotherapeutics now requires novel approaches to improve the efficiency of production. An understanding of the 3D regulatory landscape may likely give unrivaled insight into novel genome‐engineering targets capable of fine‐tuning the expression of key cellular process genes involved in cell viability, productivity, and product quality. Furthermore, a structural overview of the CHO genome is required to overcome many of the bottlenecks associated with random transgene integration, such as the effects of local chromatin organization and epigenetic repression, and to identify CHO genomic loci capable of conveying predictable and stable expression of integrated transgenes. It is our belief that the integrative map of 3D genome organization within the CHOK1SV® 10E9 cell line provided in this body of work, will, notwithstanding variation in chromatin architecture between different CHO strains and variants, be vitally important in helping to plug these knowledge gaps and will lead to improved and streamlined industrial CHO cell line development.

## 2 MATERIALS AND METHODS

### 2.1 Cell culture

CHOK1SV 10E9 cells (Zhang et al., 2015) were grown in CD‐CHO medium at 36.5°C, 140 rpm, a humidity of 85%, and a CO_2_ concentration of 5% in air (vol/vol). Each new subculture was seeded at an initial cell concentration of 0.2 × 10^6^ cells/ml on a 3 day/4 day subculture routine. All samples were taken from a 4‐day culture.

### 2.2 Hi‐C library preparation and data processing

Hi‐C libraries from 4 × 10^7^ cells fixed in a final concentration of 2% formaldehyde, were produced predominantly in accordance with that previously described (Nagano et al., 2015). Ligation was performed in a reduced reaction volume of 5 ml and libraries were amplified for seven or eight polymerase chain reaction (PCR) cycles before sequencing on an Illumina HiSeq 2500 in 50 bp paired‐end, high output mode by the Babraham Institute Next‐Generation Sequencing (NGS) Facility. Replicate sequence data were individually aligned using Bowtie (Langmead et al., 2009), and processed via the HiCUP pipeline (Wingett et al., 2015) under default parameters.

### 2.3 LACHESIS genome assembly

Replicate merged Hi‐C data informed genome assembly using the LACHESIS package (Burton et al., 2013). CHOK1SV input scaffolds were clustered into nine groups using a “minimum restriction fragment threshold” of five and a “maximum link density” of three. Scaffolds of more than 20 HindIII restriction fragments could be included within ordered trunks. Groups of low confidence ordered and/or orientated input scaffolds were manually curated into an optimal conformation, based on the appearance of artefactual long‐range interaction clusters within low‐resolution genome‐wide contact maps. The largest scaffold group was fragmented into five constituent parts based on the alignment profile to Chinese hamster chromosome sequences (Brinkrolf et al., 2013).

### 2.4 Analysis of Hi‐C data

A/B compartments were identified through principal component analysis using the HOMER software package (Lin et al., 2012), under default parameters. The coordinates of 152 actively transcribed loci were input as seed regions and upon instances when the first principal component represented the segregation of chromosome arms, data from the second principal component was used. Topologically associated domains (TADs) were also identified using the HOMER software package at a resolution of 5 kb. TAD domains were output into the *domains.bed file, the base pair extremities of these domains were defined as TAD boundaries for this study.

### 2.5 ATAC‐Seq library preparation and data processing

ATAC‐Seq was performed largely in accordance with the published protocol (Buenrostro et al., 2013), starting with ≈40,000 cells lysed for 15 min in 0.4% Igepal CA‐630 lysis buffer. Libraries were amplified for 10 PCR cycles before sequencing on an Illumina HiSeq 2500 in 50 bp paired‐end, high output mode by the Babraham Institute NGS Facility. Raw reads were processed through Trim Galore (https://www.bioinformatics.babraham.ac.uk/projects/trim_galore/) and aligned using Bowtie2 (Langmead & Salzberg, 2012) with a maximum paired‐end fragment length of 2 kb and a quality score cut‐off of 20.

### 2.6 Design of RNA bait capture system

Custom RNA baits were designed against promoter‐containing HindIII restriction fragments, that is, those encompassing the coordinates of annotated transcription start sites, using criteria previously described (Schoenfelder et al., 2015). In total, 37,973 probes spanning 21,939 promoter‐containing HindIII restriction fragments were successfully designed. The complete library of capture biotinylated RNAs was synthesized by Agilent Technologies.

### 2.7 PCHi‐C library preparation and initial data processing

Hi‐C libraries were enriched in accordance with the Agilent Technologies SureSelect Target Enrichment protocol and as previously described (Schoenfelder et al., 2015). Libraries were amplified for four or five PCR cycles, before sequencing on an Illumina HiSeq 2500 in 50 bp paired‐end, high output mode by the Babraham Institute NGS Facility. Replicate PCHi‐C sequence data were aligned and processed as per Hi‐C replicate datasets. The resulting alignments were further filtered to only include read‐pairs where at least one end mapped to a promoter containing HindIII restriction fragment captured by the RNA bait library.

### 2.8 Identifying statistically significant PCHi‐C interactions

The CHiCAGO pipeline (Cairns et al., 2016) was used to call statistically significant interactions from PCHi‐C datasets under default parameters. Interaction plots for individual baited HindIII restriction fragments were created using the plotBaits function (Cairns et al., 2016). Feature enrichments were calculated using the peakEnrichment4Features function (Cairns et al., 2016).

### 2.9 K‐means PCHi‐C interaction clustering

Statistically significant PCHi‐C interactions identified within replicate merged datasets of either cell line were filtered to remove *trans* interactions and those involving baited regions possessing more than 30 significant interactions. Baited regions possessing an inflated number of statistically significant interactions were predicted to populate low‐confidence regions of our genome assembly. Using a previously developed pipeline (Thiecke et al., 2020), CHiCAGO interaction scores were inverse hyperbolic sine transformed and capped based on a score threshold of the median plus five times the median absolute deviation. Based on biological interpretation, coupled with analysis of the variance within clusters, PCHi‐C interactions from either cell line were partitioned into five clusters.

## 3 RESULTS

### 3.1 Assembly of the highly fragmented CHOK1SV genome sequence

In an attempt to alleviate the constraints imposed by previous fragmented genome assemblies on the genome‐wide analysis of CHO chromatin structure, we first generated Hi‐C libraries, derived from the industrially‐relevant CHOK1SV 10E9 cell line (Zhang et al., 2015), and used the data to inform the assembly of CHOK1SV scaffolds initially constructed from short‐read Illumina sequences.

**Figure 1:**
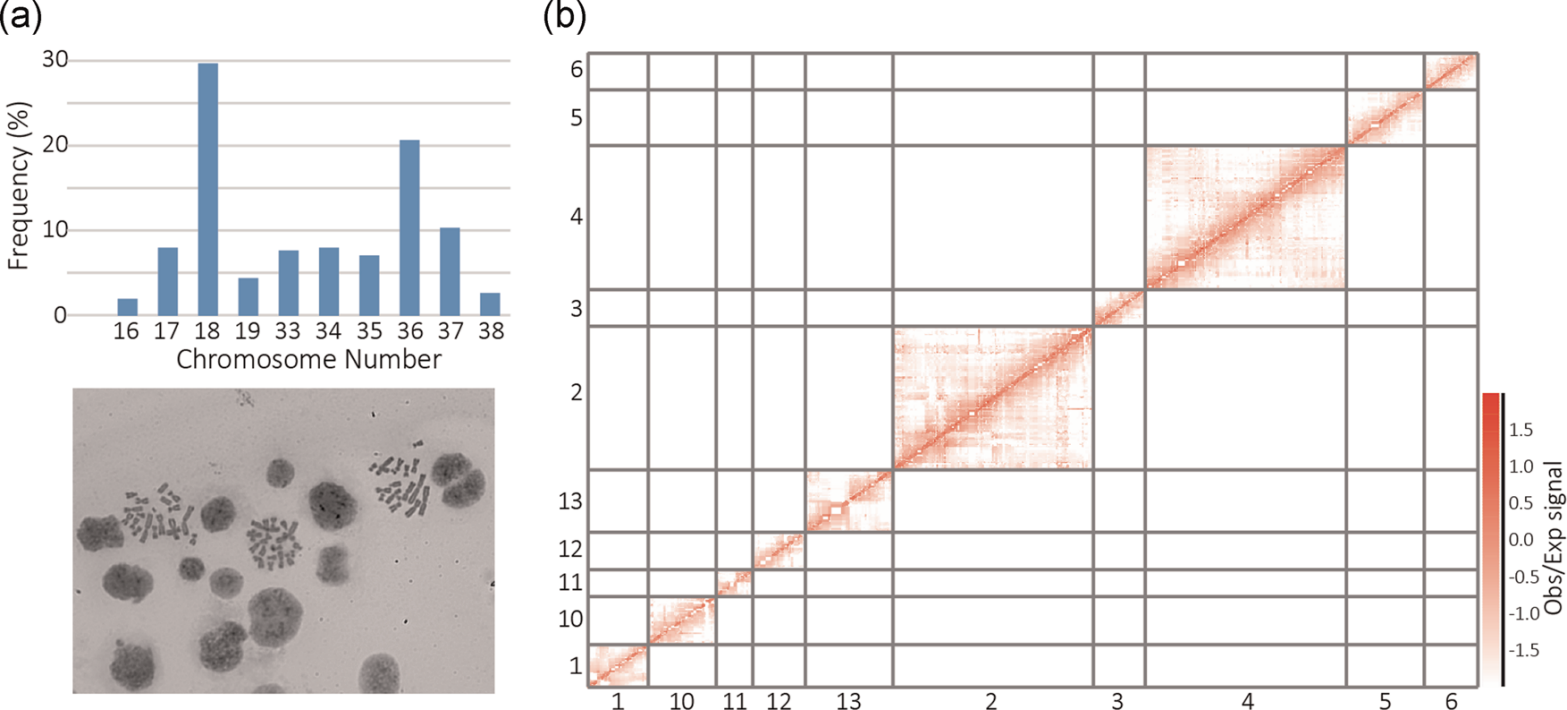
(a) The results of CHOK1SV 10E9 metaphase spread analysis (*n* = 300) coupled with an example microscopy image. (b) Section of an observed/expected Hi‐C heatmap for data aligned to the CHOK1SV 10E9 LACHESIS assembly at a resolution of CHOK1SV input scaffolds. Only *cis*‐interactions are plotted and the smallest LACHESIS assembly scaffold groups 7–9 are not included for visual clarity [Color figure can be viewed at wileyonlinelibrary.com]

Hi‐C data are characterized by an increased density of contacts between regions within the same chromosome and regions residing close to each other on the linear sequence. These characteristics have been routinely exploited to scaffold an array of fragmented genome assemblies including the recent PICRH Chinese hamster assembly (Hilliard et al., 2020). To generate the highest quality derivative CHOK1SV cell line assembly, we used over 320 million unique, valid Hi‐C read‐pair alignments from three biological replicates to cluster, order, and orientate CHOK1SV scaffolds via the LACHESIS pipeline (Burton et al., 2013). As part of this study, we confirmed that the dominant haploid chromosome number for CHOK1SV 10E9 cells was nine (Figure 1a), consistent with karyotypic analyses of other derivative CHO‐K1 cell lines adapted to glutamine free media (Vcelar et al., 2018).

To possess sufficient Hi‐C alignments to be deemed informative for assembly, a minimum restriction fragment threshold of five per input scaffold was implemented, thus resulting in 1,939 input scaffolds corresponding to ≈96% (2.17 Gb) of the total CHOK1SV sequence (Table 1). Of those 1,939 input scaffolds, 1,146, comprising 90.52% of the original sequence, were included in the final CHOK1SV 10E9 LACHESIS assembly. The final assembly consisted of 13 high confidence scaffold groups, with a length profile ranging from ≈12 Mb to ≈455 Mb (Table 1). The N50 statistic for our assembly is 175.43 Mb covered by the four largest chromosome‐scale scaffold groups (Table 1). In comparison to previous assemblies, these assembly statistics (Table 1) further exemplifies to the CHO community the power of Hi‐C mediated genome scaffolding, are consistent with those published for the recent PICRH Chinese hamster assembly (Hilliard et al., 2020) and in line with what would be expected given the haploid chromosome number of the CHOK1SV 10E9 cell line. The ≈10% of sequence not included within the final chromosome‐scale assembly remain pertinent for continued improvements to the assembly. However, it is important to note that the work presented subsequently utilizes only the chromosome‐scale CHOK1SV 10E9 LACHESIS assembly, owing to the potential issues associated with 3D structural genomic analysis of fragmented reference sequences.

**Table 1:** Derivative Chinese hamster genome assembly metrics

	Unassembled CHOK1SV input sequences^a^	CHOK1SV 10E9 LACHESIS assembly	CHO‐K1GS_HDv1 reference sequence (GCA_900186095.1)	CriGri‐PICR reference sequence (Rupp et al., 2018)
Length (Gb)	2.17	2.04	2.36	2.37
Scaffolds	1,939	13	8,264	1,830
Scaffold N50 (Mb)	4.09	175.43	62.04	19.58
Scaffold L50	158	4	12	33

^a^ Input sequences possessing at least five HindIII restriction fragments.

### 3.2 Assessment of the CHOK1SV 10E9 LACHESIS genome assembly

**Figure 2:**
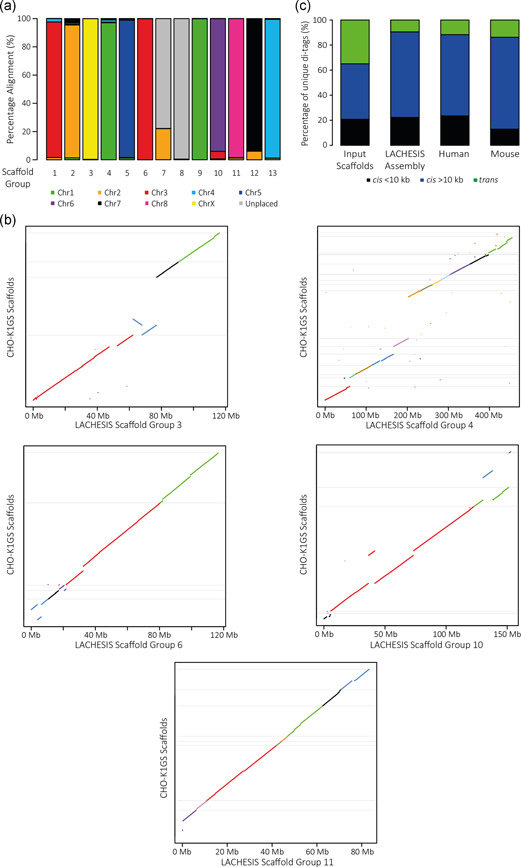
(a) Percentage alignments of each CHOK1SV 10E9 LACHESIS assembly scaffold group to sequences from individual, color‐coded Chinese hamster chromosomes (Brinkrolf et al., 2013) using a minimum alignment length of 1 kb. (b) Alignment plots for individual CHOK1SV 10E9 LACHESIS assembly scaffold groups to CHO‐K1GS_HDv1 scaffolds (GenBank Accession GCA_900186095.1). Each plot includes only those alignments for CHO‐K1GS_HDv1 scaffolds possessing a total alignment length of more than 1 Mb to the relevant chromosome‐scale scaffold group from our CHOK1SV 10E9 LACHESIS assembly. The *y*‐axis represents a pseudo linear scale corresponding to the full lengths of aligned CHO‐K1GS_HDv1 scaffolds. The minimum alignment length was set at 1 kb and alignments for CHO‐K1GS_HDv1 scaffolds in each plot are colored for visualization. (c) Stacked bar chart displaying the average percentage of unique close *cis* (<10 kb), far *cis* (>10 kb), and trans valid di‐tags across CHOK1SV 10E9 Hi‐C replicates mapped to individual input CHOK1SV scaffolds and the final CHOK1SV 10E9 LACHESIS assembly. For comparison, distributions of close *cis*, far *cis*, and trans di‐tags averaged across replicates of equivalent Hi‐C datasets derived from human embryonic stem cells and mouse fetal liver cells (Nagano et al., 2015) are included [Color figure can be viewed at wileyonlinelibrary.com]

As an initial assessment of our less fragmented assembly, we confirmed that the distinctive high proportion of close *cis*‐interactions, indicative of the increased frequency of ligation between juxtaposing regions of the linear genome, was prevalent across the diagonal of a genome‐wide Hi‐C heatmap (Figure 1b). To further validate our assembly, comparison analyses were performed against published reference sequence resources. Despite the possibility of structural rearrangements having occurred during the development of the CHOK1SV 10E9 cell line, there were clear associations between 11 of our chromosome‐scale scaffold groups (all except scaffold groups 7 and 8), and sequences from individual Chinese hamster chromosomes (Brinkrolf et al., 2013; Figure 2a). Importantly, clear alignment preferences to the same Chinese hamster chromosome (Chr 1 and Chr 3) were witnessed for two pairs of our assembled chromosome‐scale scaffold groups (Figure 2a) and should be noted as a potential avenue for future improvements to the assembly.

In comparison to the CHO‐K1GS_HDv1 reference sequence (GenBank Accession GCA_900186095.1), each CHOK1SV 10E9 LACHESIS assembly scaffold group aligned, contiguously in large sections, to a particular combination of CHO‐K1GS_HDv1 scaffolds (Figures 2b and S1). Alignment discrepancies (Figures 2b and S1) could be explained by structural rearrangements, be a result of insufficient coverage to inform optimal order/orientation, or be regions within either assembly where sequences have been incorrectly assembled. Similar levels of alignment consistency were also witnessed in comparison to the Chinese Hamster PICR reference assembly (Rupp et al., 2018; data not shown). Finally, in terms of both the overall *cis/trans* ratio and the distribution of close *cis* and far *cis*‐interactions, data aligned to the CHOK1SV 10E9 LACHESIS assembly was comparable to high‐quality human and mouse Hi‐C datasets (Nagano et al., 2015; Figure 2c).

The integration of data, such as long‐read PacBio sequencing, along with a comparative analysis to the PICRH Chinese hamster assembly (Hilliard et al., 2020), should help refine the CHOK1SV 10E9 cell line assembly further. Importantly, however, our assembly can be used to mediate the hierarchical identification of higher order 3D chromatin structures within the CHOK1SV 10E9 cell line and could be used as a framework to aid all facets of CHO cell line genomics research where a chromosome‐scale assembly is of paramount importance.

### 3.3 Uncovering endogenous CHOK1SV 10E9 higher order chromatin structures

A relationship between the 3D chromatin structure and transcription has been well documented across many cell types (Deng et al., 2012; Javierre et al., 2016; Lupiáñez et al., 2015; Reviewed in Schoenfelder & Fraser, 2019). With a substantially less fragmented CHOK1SV 10E9 genome assembly, we were in a position to interrogate these higher order structural features of the CHOK1SV 10E9 genome.

**Figure 3:**
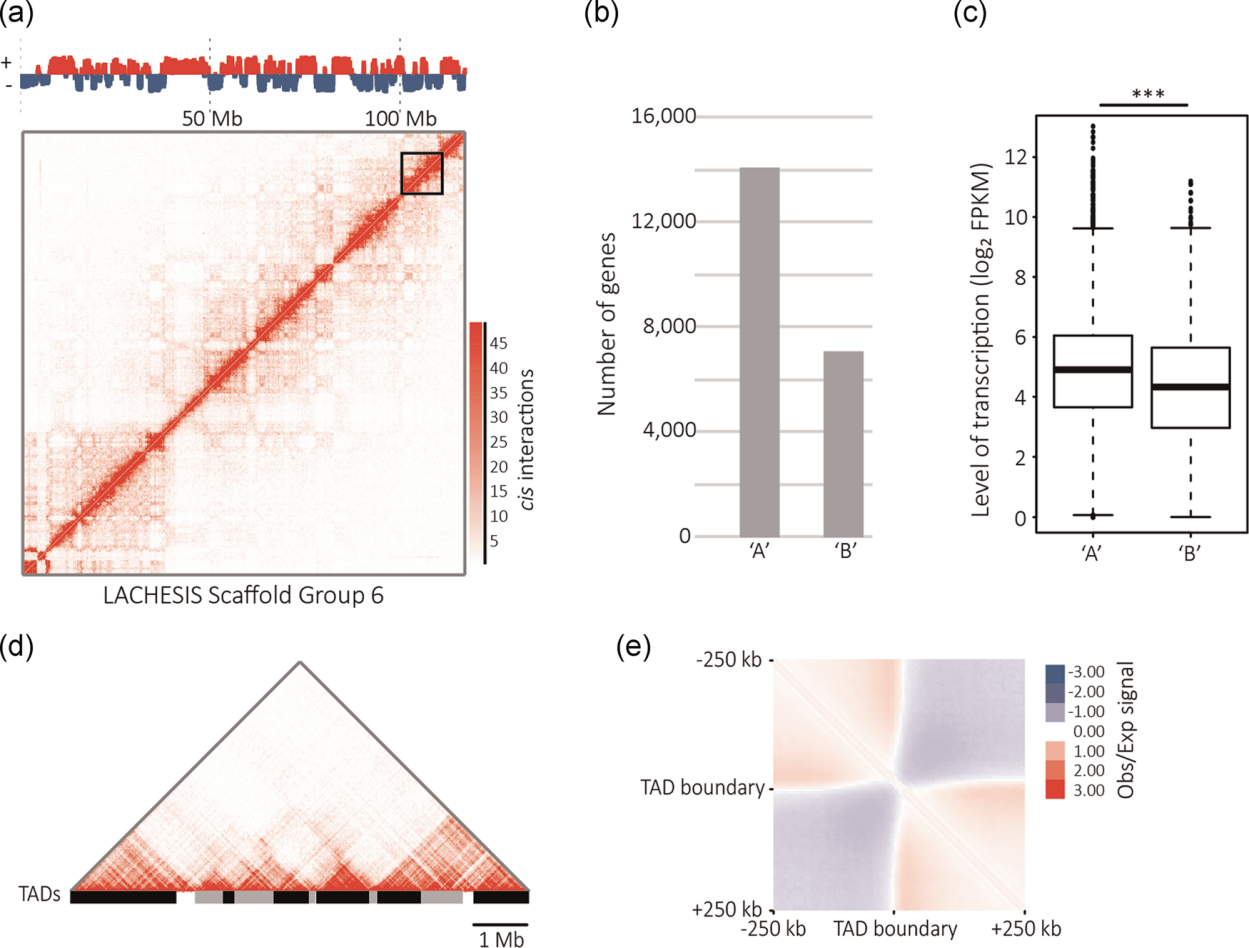
(a) The results of the principal component analysis for scaffold group 6 at a resolution of 50 kb. Bins colored in red possess a positive principal component value and are designated as “A” compartment. Bins colored in blue possess a negative principal component value and are designated as “B” compartment. The *cis*‐interaction heatmap of scaffold group 6, produced using replicate merged Hi‐C data at a resolution of 50 kb, is included. (b) Number of annotated genes contained within active “A” and inactive “B” genomic compartments. (c) Steady‐state mRNA levels for annotated genes with a log_2_ FPKM value more than 0 contained within active “A” and inactive “B” genomic compartments. Statistical significance was determined by a Wilcoxon rank‐sum test. (d) Section of the *cis*‐interaction heatmap for scaffold group 6 produced at a resolution of 5 kb with topologically associated domains (TADs), identified using replicate merged Hi‐C data, illustrated beneath. (e) Pooled genome‐wide Hi‐C contact matrix for all 500‐kb regions centered on TAD boundaries normalized for the number of expected counts [Color figure can be viewed at wileyonlinelibrary.com]

Through principal component analysis of our Hi‐C data, we identified active (“A”) and inactive (“B”) genomic compartments within our chromosome‐scale assembly (Figure 3a). On average, active and inactive genomic compartments were 1.36 Mb in length and were equally distributed with active “A” compartments spanning 49.7% and inactive “B” compartments 50.3% of the assembly. “A” compartments overlap a large proportion of annotated genes (Figure 3b) whose expression is significantly higher in comparison to genes enclosed in the more transcriptionally inactive “B” compartments (Figure 3c). We next used the Hi‐C data to establish the confines of 3,644 endogenous TADs (Illustrated in Figure 3d), covering over 85% of the assembly with a mean domain length of 478 kb. As expected, we detected a strong preference for intradomain interactions (Figure 3e). Thus, our data reveal important 3D structural features of the CHOK1SV 10E9 genome.

### 3.4 Using chromatin accessibility data to inform an enhanced ChromHMM model

**Figure 4:**
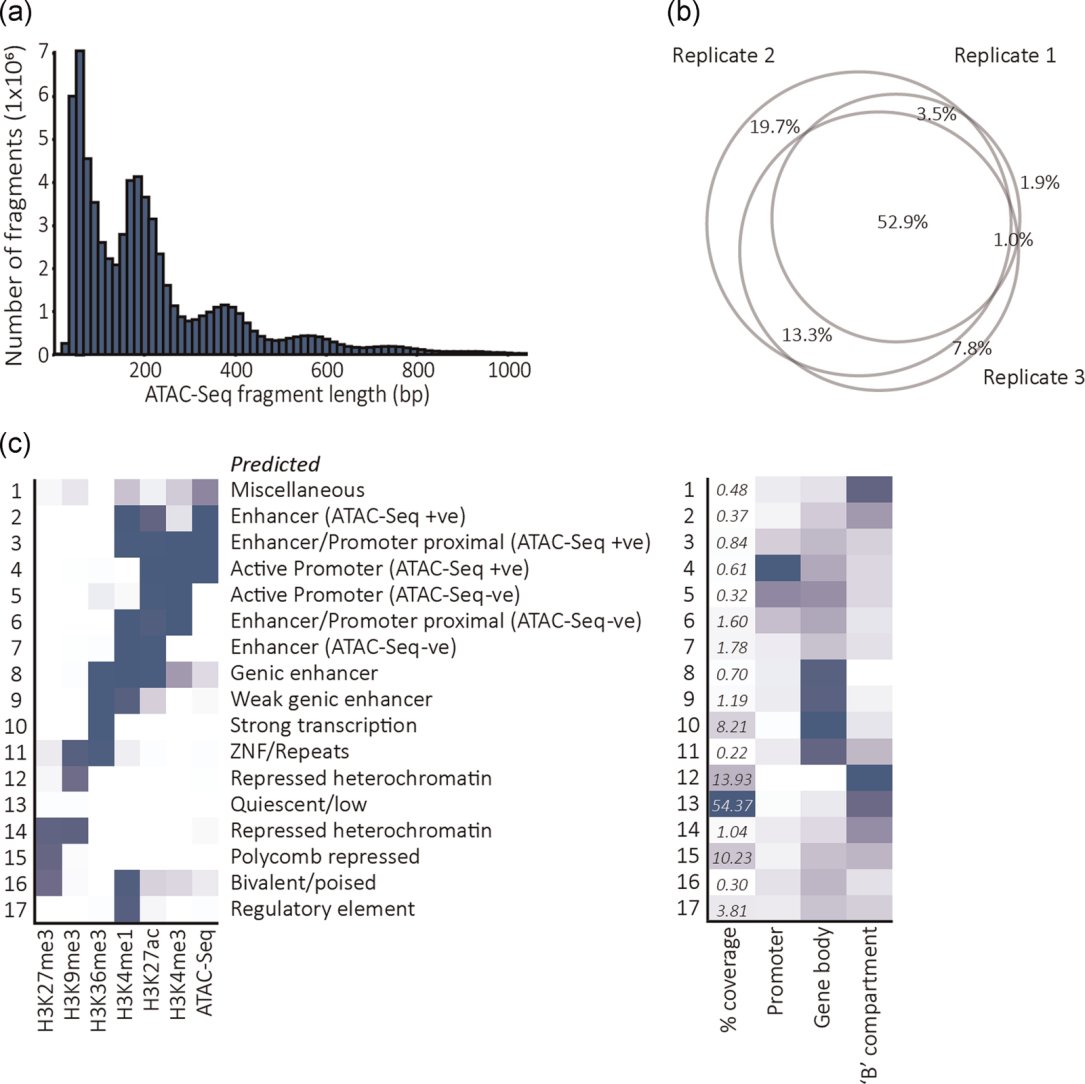
(a) Fragment length profile for sequenced CHOK1SV 10E9 ATAC‐Seq fragments aligned to the CHOK1SV 10E9 LACHESIS chromosome‐scale assembly. (b) Venn diagram illustrating the consistency in peak chromatin accessibility across three biological replicates. The total peak number represents the union of peaks present in any of the three CHOK1SV 10E9 replicate datasets. (c) (Left) Heatmap illustrating the distribution of input marks across the 17‐state ChromHMM model. The model was informed by H3K4me1, H3K4me3, H3K9me3, H3K36me3, H3K27me3, and H3K27ac published ChIP‐Seq data (Feichtinger et al., 2016) and ATAC‐Seq data from the CHOK1SV 10E9 cell line generated in this study. (Right) Heatmap illustrating the overlaps of individual ChromHMM states with specific genomic features, principally 1‐kb promoter windows upstream of annotated transcription start sites, annotated gene bodies, and inactive “B” compartments defined through principal component analysis of our Hi‐C data [Color figure can be viewed at wileyonlinelibrary.com]

We next used ATAC‐Seq (Buenrostro et al., 2013) to identify accessible regions of the CHOK1SV 10E9 genome. The accessibility of chromatin is an attribute correlated with transcriptional activity. In total, 68 million valid read‐pairs, conforming to the expected ≈150 bp periodicity of fragment sizes (Figure 4a), informed the identification of over 37,000 highly reproducible (present in all three replicates) regions of accessible chromatin (Figure 4b). We integrated our ATAC‐Seq data with published histone modification ChIP‐Seq data (Feichtinger et al., 2016) to inform a comprehensive 17‐state ChromHMM (Ernst & Kellis, 2012) model that builds upon the functional annotation of the CHO genome (Feichtinger et al., 2016; Figure 4c). As expected, a clear overlap between ATAC‐Seq signal and states representative of active promoters and enhancers was observed (Figure 4c). In contrast, states enriched in the repressive heterochromatic mark H3K9me3 predominantly overlapped our inactive “B” compartments (Figure 4c).

Including chromatin accessibility within the ChromHMM model should, in theory, refine the location of predicted active enhancer sequences beyond that possible using the canonical signature of histone modifications. Taking candidate active enhancers defined as the union of ChromHMM states 2, 3, 6, and 7, all of which correspond to the canonical active enhancer histone modification signature (H3K4me1 and H3K27ac), we found that 43.5% of them overlap accessible (ATAC‐Seq positive) enhancer regions. Across these regions, signal from ATAC‐Seq positive enhancer states (2 and 3) dominate the central regions whereby the ATAC‐Seq negative enhancer states (6 and 7) correspond to signal generated by flanking nucleosomes (Figure S2a). A similar pattern is uncovered at promoter regions where the ATAC‐Seq positive promoter‐associated signal (State 4) predominantly overlaps the sequences immediately upstream of transcription start sites (Figure S2b).

### 3.5 Probing the 3D promoter interactome

We subsequently employed PCHi‐C (Schoenfelder et al., 2015) and the CHiCAGO analysis pipeline (Cairns et al., 2016), to uncover, at high resolution, chromatin interactions that connect gene promoters with other regulatory elements including enhancers. The functionality of CHiCAGO relies upon a genome‐wide background model to normalize for distance‐dependent and technical noise (Cairns et al., 2016). Further corroborating our improved CHOK1SV 10E9 genome assembly, diagnostic plots, and many bait‐specific interaction plots conformed to the expected trends (Cairns et al., 2016; Figures S3 and S4).

**Figure 5:**
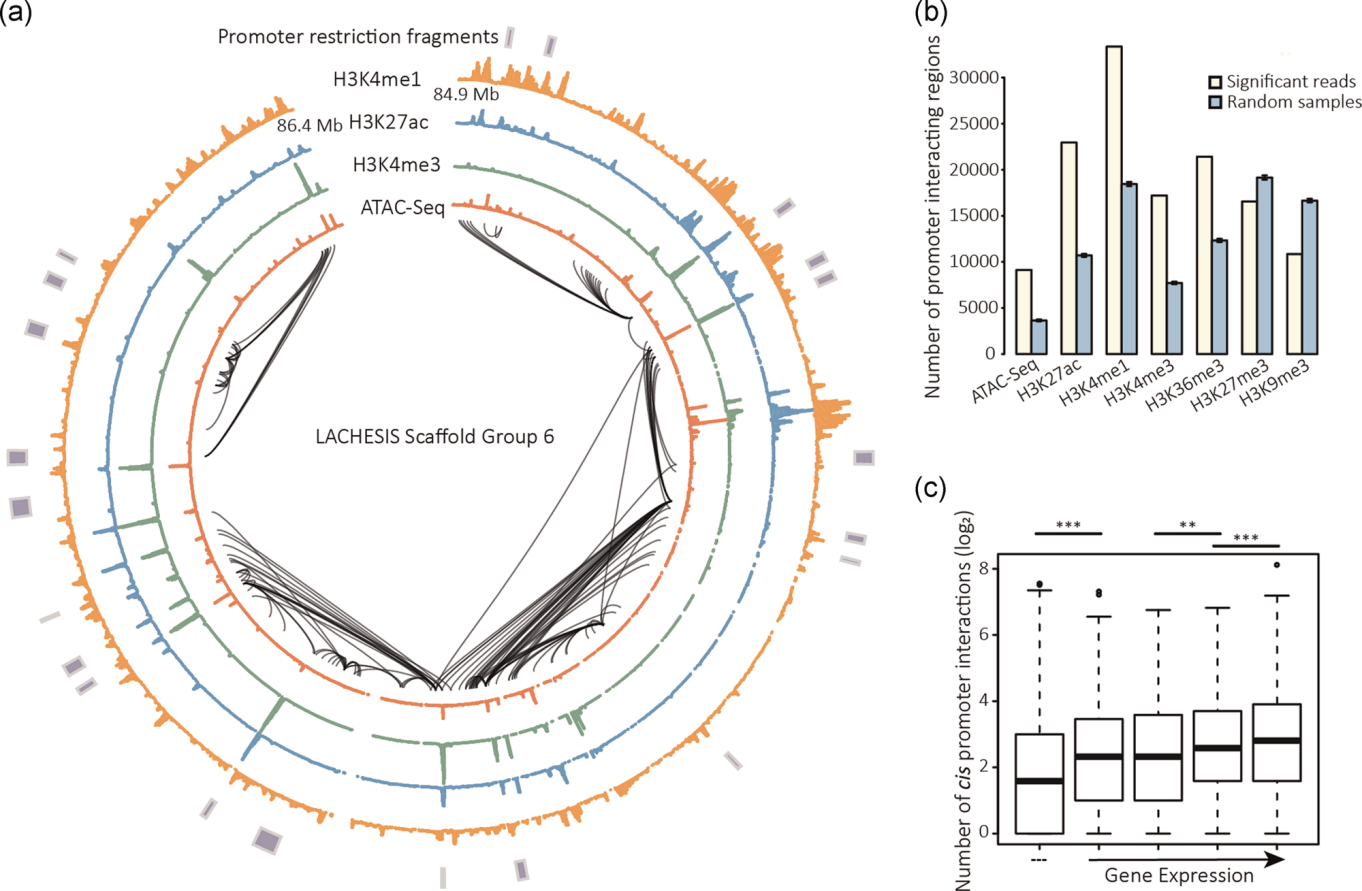
(a) Circos plot illustrating statistically significant promoter interactions across a 1.5‐Mb region of scaffold group 6. Annotated are the H3K4me1, H3K27ac, H3K4me3, and ATAC‐Seq signals across the same region along with the location of promoter HindIII restriction fragments for which interactions were enriched. (b) Statistically significant promoter interacting regions enriched for peaks in active histone modifications and chromatin accessibility. The null hypothesis represents the overlap expected by chance from random samples of promoter interacting regions matched for the overall distribution of interaction distances. (c) Average number of statistically significant *cis*‐interactions per promoter possessing at least one statistically significant interaction, split into expression categories based on steady‐state mRNA levels. Statistical significance was determined by a Wilcoxon rank‐sum test [Color figure can be viewed at wileyonlinelibrary.com]

In total, we identified 107,331 high confidence, *cis*‐promoter contacts within CHOK1SV 10E9 nuclei (Illustrated in Figure 5a), with a median number of four interactions per promoter fragment and a median interaction distance of ≈141 kb. Approximately, 17% of interactions are between two promoters with a median interaction distance of 119.5 kb. In total, we identified 57,947 nonpromoter interacting regions of which 12.1% interact with at least three promoter fragments. Consistent with previous mouse and human PCHi‐C datasets (Javierre et al., 2016; Mifsud et al., 2015; Schoenfelder et al., 2015), these promoter interacting regions were enriched for histone modifications associated with active chromatin such as H3K4me1, H3K4me3, H3K27ac, and H3K36me3 (Figure 5b). Furthermore, we found that gene expression in CHOK1SV 10E9 cells is positively correlated with the number of *cis*‐interactions per promoter, an observation also reported in human and mouse cell types (Javierre et al., 2016; Schoenfelder et al., 2015; Figure 5c). Interestingly, accessible, active enhancer regions involved in these significant promoter interactions were enriched for the binding motif of CTCF (*p* = 1 × 10^‐78^) over those accessible, active enhancer regions not identified as promoter interacting in this study. This is consistent with the concept that CTCF is involved in the formation and maintenance of chromatin loop interactions between functional regulatory elements (Dowen et al., 2014; Rao et al., 2014). Of the ≈92% of high confidence, *cis*‐promoter contacts where both interacting fragments could be unequivocally assigned to a single TAD or a single inter‐TAD region, we found that 20.4% of contacts span across a TAD boundary. This is broadly consistent with what has been witnessed across many human cell types (Javierre et al., 2016) and reaffirms our identification of TADs within the CHOK1SV 10E9 genome.

In the context of cell line engineering, the identification of these functional contacts could be important in fine‐tuning the expression of genes whose reduced or increased expression can have a positive effect on product titer and/or product quality. Candidate enhancers identified by this study to interact with the gene encoding the pioneer transcription factor *Foxa1*, overexpression of which has been shown to increase transgenic protein production (Berger et al., 2020), and the gene encoding the sialidase *Neu2*, could in the future be targets of interest (Figure S5).

### 3.6 An improved understanding of chromatin architecture can underpin improved phenotypic output

To investigate the power of our work, we set out to understand the architectural differences between two clonal cell lines. Our model CHOK1SV 10E9 cell line possesses a predefined landing pad within the *Fer1l4* locus created using FLP/FRT recombinase‐mediated cassette exchange (Zhang et al., 2015). This locus was identified following extensive analysis of randomly integrated clones and was shown to accommodate high‐level productivity and long‐term stability of integrated transgenes (Zhang et al., 2015). We generated ATAC‐Seq and PCHi‐C datasets from the CHOK1SV A8A10 cell line, a derivative of the CHOK1SV 10E9 cell line, in which a monoclonal antibody construct is integrated and expressed from within the *Fer1l4* genomic locus (Zhang et al., 2015).

**Figure 6:**
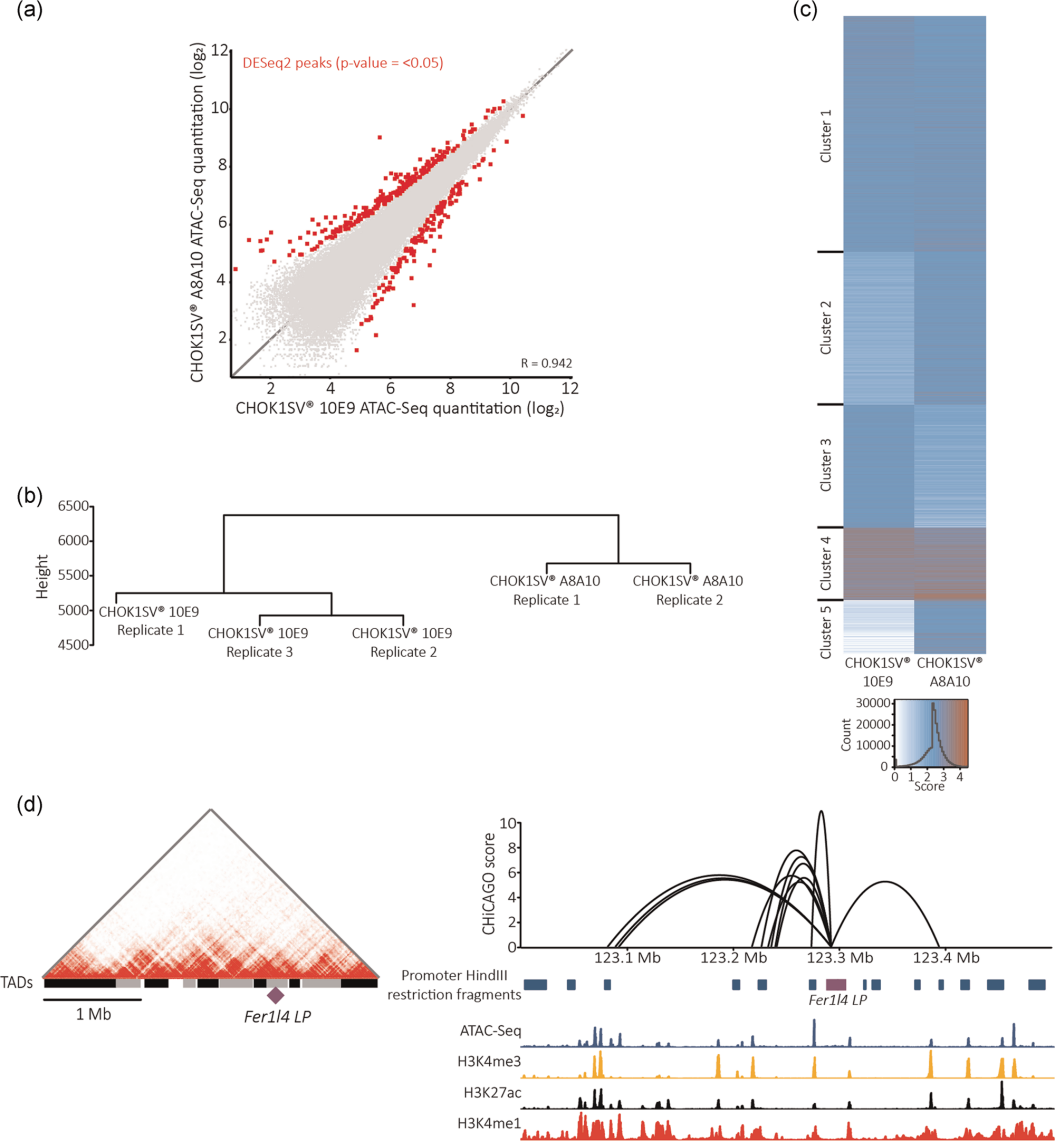
(a) Scatterplot of quantitated ATAC‐Seq peak regions called from any replicate of our two experimental cell lines. Quantitation represents normalized, log_2_ transformed fragment counts. Highlighted are differential ATAC‐Seq peak regions identified by DESeq2 with a *p* = < .05. (b) PCHi‐C interaction profile dendrogram for all replicates of the two experimental cell lines. (c) Heatmap of all PCHi‐C interactions clustered via a nonthreshold K‐means clustering approach (Thiecke et al., 2020). Interaction scores are color‐coded on a gradient from white to red. Cluster 5 represents the cluster of CHOK1SV A8A10 cell line‐specific interactions possessing a much stronger signal in comparison to the CHOK1SV 10E9 cell line. (d) Structural characteristics for the industrially relevant *Fer1l4* landing pad (Zhang et al., 2015; purple). (Left) Location of the locus with respect to TADs identified in the vicinity. (Right) Interaction profile of the locus annotated with the locations of baited, promoter HindIII restriction fragments, and ATAC‐Seq, H3K4me3, H3K27ac, and H3K4me1 signal quantitated across overlapping 500‐bp windows [Color figure can be viewed at wileyonlinelibrary.com]

In comparison to the map of chromatin organization previously defined for the CHOK1SV 10E9 cell line, the vast majority of accessible sites were consistent between the two experimental cell lines; however, 383 regions exhibited differential accessibility (*p* =  <.05; Figure 6a). Furthermore, through analysis of the 3D interactome, we find clear segregation between the chromatin interaction profiles of the two cell lines (Figure 6b). To resolve the differences in interaction profile further, a nonthreshold K‐means clustering approach, based on the interaction significance scores determined by CHiCAGO, was undertaken as previously described by Thiecke et al. (2020). Through this analysis, four of the defined clusters represented interactions with similar significance scores in both cell lines; however, a cluster of 9,132 statistically significant, *cis*‐promoter interactions enriched in the antibody expressing CHOK1SV A8A10 cell line was identified (Cluster 5; Figure 6c). This cluster included interactions for 792 captured HindIII restriction fragments totaling 567 functionally annotated gene promoters not represented by interactions in any of the other four clusters. An intrinsic variability between the individual clones used to derive the experimental cell lines is likely to contribute to the subtle differences in chromatin organization. Despite this, however, genes encoding the eukaryotic initiation factors *Eif3H* and *EIf4a3*, nuclear pore complex proteins *Pom121* and *Nup43*, the Golgi‐associated protein *Golga7*, and numerous ribosomal proteins are present within the subset of functionally annotated gene promoters solely possessing CHOK1SV A8A10 specific interactions. We, therefore, hypothesize that these newly formed interactions are related to the requirements of high‐level transgene protein production within the CHOK1SV A8A10 cell line. Importantly, even with an increased cluster number of seven, a corresponding cluster enriched in CHOK1SV 10E9 specific interactions was not observed (data not shown).

The identification of CHO 3D genome organization will help to form a comprehensive platform for the improved identification of novel genome‐engineering target sites and to benchmark the regulatory landscape of known genome‐engineering sites. With relevance to this, we find that the *Fer1l4* genomic locus resides within an active compartment approximately 70 kb from the periphery of a TAD spanning 215 kb and makes contacts with a number of expressed gene promoters within the flanking 400 kb (Figure 6d). As it is known through empirical analysis that this locus conveys high‐level transgene production as well as an inherent level of production stability (Zhang et al., 2015), it is reassuring that we find the 3D structural and regulatory landscape of this locus to also be associated with transcriptional activity. This is an exemplar analysis of an individual locus and we anticipate that our map of 3D chromatin architecture will facilitate and can be relied upon to aid in the discovery of additional, much needed, novel “safe harbor” loci.

## 4 DISCUSSION

As the requirements placed upon the biopharmaceutical industry continue to grow, a deeper understanding of CHO biology and in particular, its effect on cell line performance has become even more critical. Given the level of flexibility required to produce the wide range of complex biotherapeutics now industrially produced in CHO cells, it is highly desirable to develop the tools and increase the capability for rational cell line and vector engineering. Recombinant CHO cell lines used by the biopharmaceutical industry are hampered by issues such as unpredictable expression levels and transgene silencing. There is a currently unmet need within the biotechnology sector to identify endogenous integration sites that confer consistent, predictable, and stable expression of integrated payloads. With the gene‐editing tools now available, an understanding of the endogenous 3D chromatin conformation is essential to reduce the theoretical blank canvas of genome‐engineering targets to a shortlist of desirable candidates. Facilitated by a significantly less fragmented CHOK1SV 10E9 genome assembly, our identification of higher order chromatin structures, spatially segregating the CHOK1SV 10E9 genome based on transcriptional activity, immediately aids in this quest. Importantly, regions in close proximity to TAD boundaries are known to be enriched in expressed housekeeping genes and in factors linked to active transcription (Dixon et al., 2012). An increased level of genomic and transcriptional stability predicted to result from this proximity to vital housekeeping genes, could result in these regions being interesting targets for site‐specific transgene integration.

Aside from the requirements for transgene integration sites, flexibility in terms of recombinant cell line phenotypes, for example, cell lines with an increased capacity for protein translation, increased bioreactor cell viability, or tailored protein glycosylation patterns, would also be highly beneficial for the biopharmaceutical sector. Recent exemplar research has uncovered the positive effects of ACTC1 (Pourcel et al., 2020) and SCD1 (Budge et al., 2020) overexpression on not only transgene production but also in reducing the toxic effects of lactate metabolism and expanding the endoplasmic reticulum, respectively. A key part of this study is our production of a CHOK1SV 10E9 3D genome interactome, identifying the active distal regulatory elements that interact in 3D space with, and influence the transcription of, specific target genes. Leveraging this information could fine‐tune the expression of candidate genes resulting in the “toolbox” of recombinant CHO cell lines necessary for optimal production of the wide range of bespoke complex biotherapeutics (O'Callaghan et al., 2015).

We acknowledge that the variability in the endogenous chromatin architecture of clonal CHO cell lines, pertaining to general chromatin dynamics and the inherent genetic instability of immortalized CHO cell lines, will be an important factor to consider when relating this data to other strains and/or variants. Taken together, however, our in‐depth integrative map of 3D genome organization and chromatin accessibility within an industrially relevant clonal CHO host cell line provides a further important advancement for CHO cell line development and acts as a tool for the improved identification and predictive assessment of genome engineering targets. We predict that the scope of potential genome engineering targets informed by a much greater understanding of the endogenous 3D chromatin architecture will translate into the development of an improved, more streamlined recombinant protein production system within industry, leading to a quicker transition of novel therapeutics into the market. In addition, as the field of CHO cell line development continues to evolve, the knowledge and resources provided here will continue to be highly beneficial and relevant for biotechnological applications for the foreseeable future.

## CONFLICT OF INTERESTS

Peter Fraser and Stefan Schoenfelder and are cofounders of Enhanc3D Genomics Ltd. The other authors declare that there are no conflict of interests.

## AUTHOR CONTRIBUTIONS

Peter M. O'Callaghan and Peter Fraser conceived the project and the main conceptual ideas. Stephen Bevan, Peter M. O'Callaghan, Peter Fraser, Robert J. Young, Lin Zhang, and Stefan Schoenfelder contributed to experimental design. Stephen Bevan performed Hi‐C, PCHi‐C, and ATAC‐Seq experiments and analysis. Stefan Schoenfelder provided training and assistance with Hi‐C and PCHi‐C experiments. Simon Andrews designed the PCHi‐C RNA bait library and provided bioinformatics support, tools, and assistance. Peter M. O'Callaghan, Peter Fraser, Robert J. Young, Lin Zhang, and Stefan Schoenfelder supervised the research. Stephen Bevan drafted the manuscript with input from all authors. All authors provided critical feedback and contributed to the analysis and interpretation of the results.

## Supporting information

Supporting information.

Supporting information.

Supporting information.

Supporting information.

Supporting information.

Supporting information.
