# Supplementary material for: High‐resolution three‐dimensional chromatin profiling of the Chinese hamster ovary cell genome
Source: Biotechnol Bioeng. 2020 Nov 20;118(2):784–96. doi: 10.1002/bit.27607 (PMC7894165; doi:10.1002/bit.27607)
Supplement: Supplementary file 1 — Supporting information. [file BIT-118-784-s001.docx]

**Supplementary Figure 1**

Alignment plots for individual CHOK1SV® 10E9 LACHESIS assembly scaffold groups to CHO-K1GS_HDv1 scaffolds (GenBank Accession GCA_900186095.1). Each plot includes only those alignments for CHO-K1GS_HDv1 scaffolds possessing a total alignment length of >1 Mb to the relevant chromosome-scale scaffold group from our CHOK1SV® 10E9 LACHESIS assembly. The y-axis represents a pseudo linear scale corresponding to the full lengths of aligned CHO-K1GS_HDv1 scaffolds. The minimum alignment length was set at 1 kb and alignments for CHO-K1GS_HDv1 scaffolds are coloured for visualisation.

**Supplementary Figure 2**

Probe trend plots for a) the relative ATAC-Seq positive enhancer (ChromHMM states 2 and 3) and ATAC-Seq negative enhancer (ChromHMM states 6 and 7) signals across candidate enhancer regions. b) the relative ATAC-Seq positive promoter (ChromHMM state 4) and ATAC-Seq negative promoter (ChromHMM state 5) signals across 1 kb promoter windows upstream of transcription start sites (TSS).

**Supplementary Figure 3**

1. Distance decay model calculated for the replicate merged CHOK1SV® 10E9 PCHi-C dataset following the removal of non-captured Hi-C read-pairs.
2. Brownian noise correction model for pools of interacting regions (red) and baits (blue) with increasing numbers of *trans* chromosomal interactions, again within the replicate merged CHOK1SV® 10E9 PCHi-C dataset following the removal of non-captured Hi-C read-pairs. PIR = promoter interacting region.
3. Technical noise estimates per bait pool (top) and per interacting region pool (below) for increasing numbers of *trans*-chromosomal interactions, again within the replicate merged CHOK1SV® 10E9 PCHi-C dataset following the removal of non-captured Hi-C read-pairs.

**Supplementary Figure 4**

Bait specific interaction profiles illustrating raw interaction read counts across a 1 Mb region centralised on the baited HindIII restriction fragment in question. Statistically significant interactions with a CHiCAGO score greater than five are illustrated in red. Interactions with a score greater than three are illustrated in blue.

**Supplementary Figure 5**

Promoter interaction profiles for a) *Foxa1* and b) *Neu2* annotated with the locations of baited, promoter HindIII restriction fragments and ATAC-Seq, H3K4me3, H3K27ac and H3K4me1 signal quantitated across overlapping 500 bp windows. Candidate enhancer interactions are boxed.

Supplementary Methods

**Karyotyping**

Cell samples were incubated for 2 hours in colcemid to a final concentration of 0.08 µg/mL. Pelleted cells were incubated at 37°C in pre-warmed 8 g/L sodium citrate hypotonic solution for 5 minutes prior to fixation in 4 mL Carnoy’s fixative.

**CHOK1SV® LACHESIS assembly gene annotations**

A complete set of gene annotation predictions, aligned to a previous iteration of CHOK1SV® sequence scaffolds, had been previously carried out using a combination of in-house RNA-Seq datasets, experimental data from Rupp et al., 2014, along with publicly available annotations including from the Chinese hamster and CHO-K1 genomes. This set of annotations included 25,648 annotated gene loci and was used to design the PCHi-C RNA bait library with each bait annotated with the relevant unique annotation ID. With regards to the CHOK1SV® LACHESIS assembly presented here, PCHi-C RNA bait sequences were uniquely aligned to confirm the identity of promoter containing HindIII restriction fragments. Gene annotations were uniquely aligned to the CHOK1SV® LACHESIS assembly by taking the top blastn alignment with e < 0.01 and using a minimum sequence alignment threshold of greater than 90% of the query length.

**Analysis of RNA-Seq data**

Triplicate total RNA-Seq paired-end datasets from the CHOK1SV® 10E9 cell line sampled on day 4 of culture were processed through Trim Galore (https://www.bioinformatics.babraham.ac.uk/projects/trim_galore/) and aligned to a previous iteration of CHOK1SV® scaffolds using HiSat2 in paired-end mode with a mapping quality score filter of 40 (Kim, Langmead, & Salzberg, 2015). Replicate datasets were merged and log_2_ FPKM values for each unique annotated gene ID were calculated using paired-end fragments that overlap annotated exons normalised for transcript length. Gene expression categories were defined based on these log_2_ FPKM values, with all genes possessing a negative value placed into a single category.

**ATAC-Seq peak calling**

Peak regions were identified using the MACS2 peak caller (Zhang et al., 2008) with the following parameters; --f BAM --g 2000000000 -q 0.01 --nolambda --nomodel --call-summits. Unions of peak regions were calculated using the GenomicRanges package (Lawrence et al., 2013) with a minimum overlap of 1 bp. Differential analysis was performed using DESeq2 (Love, Huber, & Anders, 2014) on the union of all peak regions regardless of cell line with multiple testing correction applied.

**Analysis of ChIP-Seq data**

Published single-end ChIP-Seq sequence data (Feichtinger et al., 2016) was processed through Trim Galore (https://www.bioinformatics.babraham.ac.uk/projects/trim_galore/) and mapped to the CHOK1SV® 10E9 LACHESIS assembly using Bowtie2 (Langmead & Salzberg, 2012) with a maximum fragment length of 1 kb and a quality cut-off of 20. Datasets corresponding to different time points of the same modification were merged. Peaks were identified using the MACS2 peak caller (Zhang et al., 2008) with the following parameters; -q 0.01, -g 2000000000 –nolambda –nomodel. Narrow peaks were identified for histone modifications H3K4me3 and H3K27ac. For all other modifications, broad peaks were identified.

**Construction of the 17-state ChromHMM model**

Replicate merged ATAC-Seq and time point merged ChIP-Seq BAM files informed 200bp genome wide binary enrichments using the BinarizeBed ChromHMM function (Ernst & Kellis, 2012). A 17-state ChromHMM model was derived using the LearnModel function (Ernst & Kellis, 2012).

**Motif Analysis**

Motif analysis was performed using the HOMER software package (Heinz et al., 2010).

Supplementary References

Ernst, J., & Kellis, M. (2012). ChromHMM: automating chromatin-state discovery and characterization. *Nature Methods*, Vol. 9, pp. 215–216. doi: 10.1038/nmeth.1906

Feichtinger, J., Hernández, I., Fischer, C., Hanscho, M., Auer, N., Hackl, M., … Borth, N. (2016). Comprehensive genome and epigenome characterization of CHO cells in response to evolutionary pressures and over time. *Biotechnology and Bioengineering*, *113*(10), 2241–2253. doi: 10.1002/bit.25990

Heinz, S., Benner, C., Spann, N., Bertolino, E., Lin, Y. C., Laslo, P., … Glass, C. K. (2010). Simple combinations of lineage-determining transcription factors prime cis-regulatory elements required for macrophage and B cell identities. *Molecular Cell*, *38*(4), 576–589. doi: 10.1016/j.molcel.2010.05.004

Kim, D., Langmead, B., & Salzberg, S. L. (2015). HISAT: a fast spliced aligner with low memory requirements. *Nature Methods*, *12*(4), 357–360. doi: 10.1038/nmeth.3317

Langmead, B., & Salzberg, S. L. (2012). Fast gapped-read alignment with Bowtie 2. *Nature Methods*, *9*(4), 357–359. doi: 10.1038/nmeth.1923

Lawrence, M., Huber, W., Pagès, H., Aboyoun, P., Carlson, M., Gentleman, R., … Carey, V. J. (2013). Software for computing and annotating genomic ranges. *PLoS Computational Biology*, *9*(8), e1003118. doi: 10.1371/journal.pcbi.1003118

Love, M. I., Huber, W., & Anders, S. (2014). Moderated estimation of fold change and dispersion for RNA-seq data with DESeq2. *Genome Biology*, *15*(12), 550. doi: 10.1186/s13059-014-0550-8

Rupp, O., Becker, J., Brinkrolf, K., Timmermann, C., Borth, N., Pühler, A., … Goesmann, A. (2014). Construction of a public CHO cell line transcript database using versatile bioinformatics analysis pipelines. *PloS One*, *9*(1), e85568. doi: 10.1371/journal.pone.0085568

Zhang, Y., Liu, T., Meyer, C. A., Eeckhoute, J., Johnson, D. S., Bernstein, B. E., … Liu, X. S. (2008). Model-based analysis of ChIP-Seq (MACS). *Genome Biology*, *9*(9), R137. doi: 10.1186/gb-2008-9-9-r137
