# Supplementary figures and images for: High‐resolution three‐dimensional chromatin profiling of the Chinese hamster ovary cell genome
Source: Biotechnol Bioeng. 2020 Nov 20;118(2):784–96. doi: 10.1002/bit.27607 (PMC7894165; doi:10.1002/bit.27607)

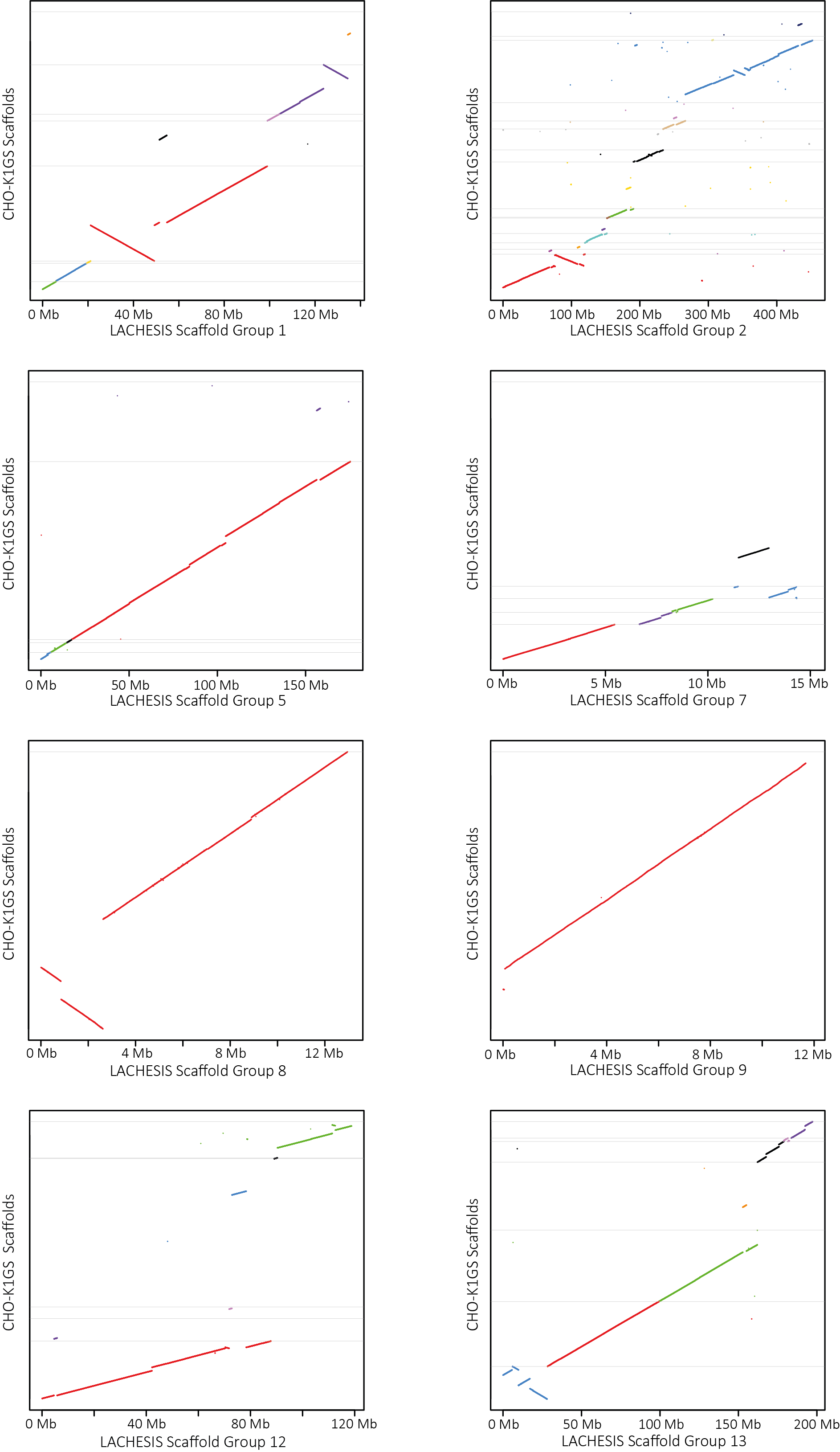

Supplement: Supplementary file 2 — Supporting information. [file BIT-118-784-s002.png]

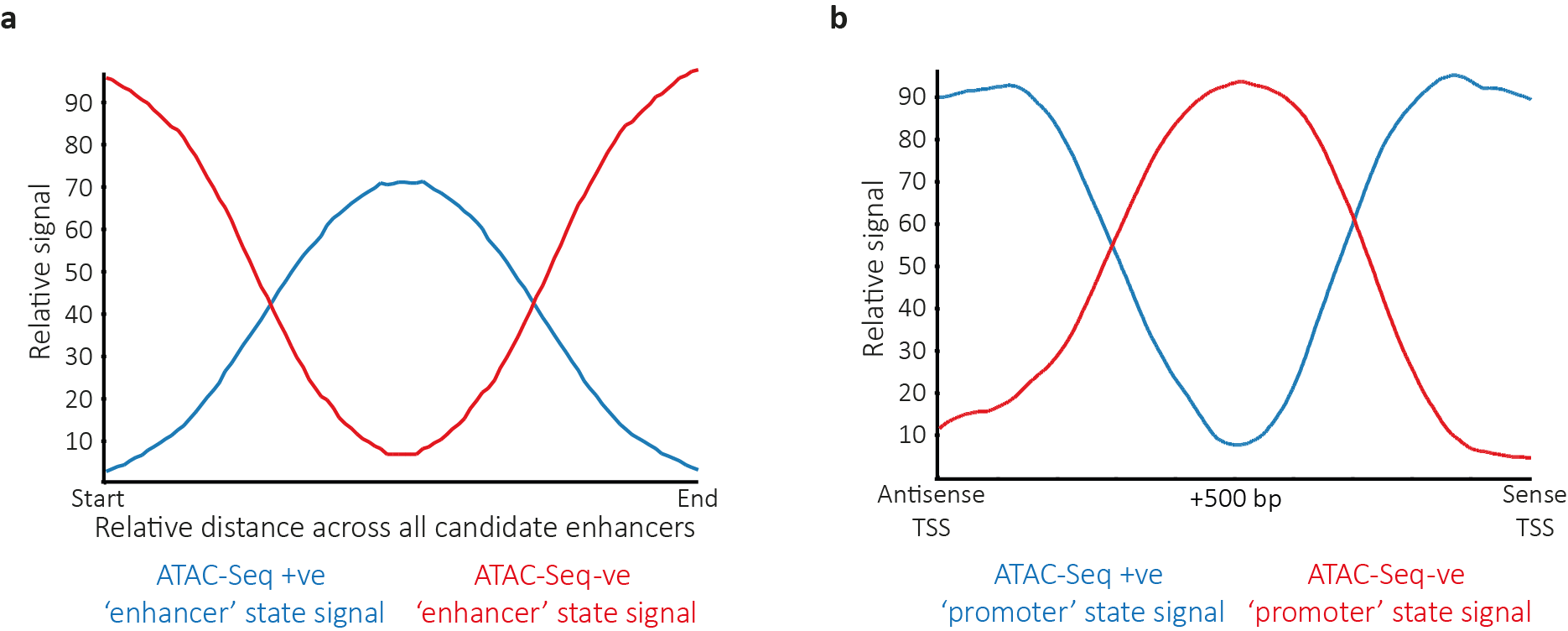

Supplement: Supplementary file 3 — Supporting information. [file BIT-118-784-s003.png]

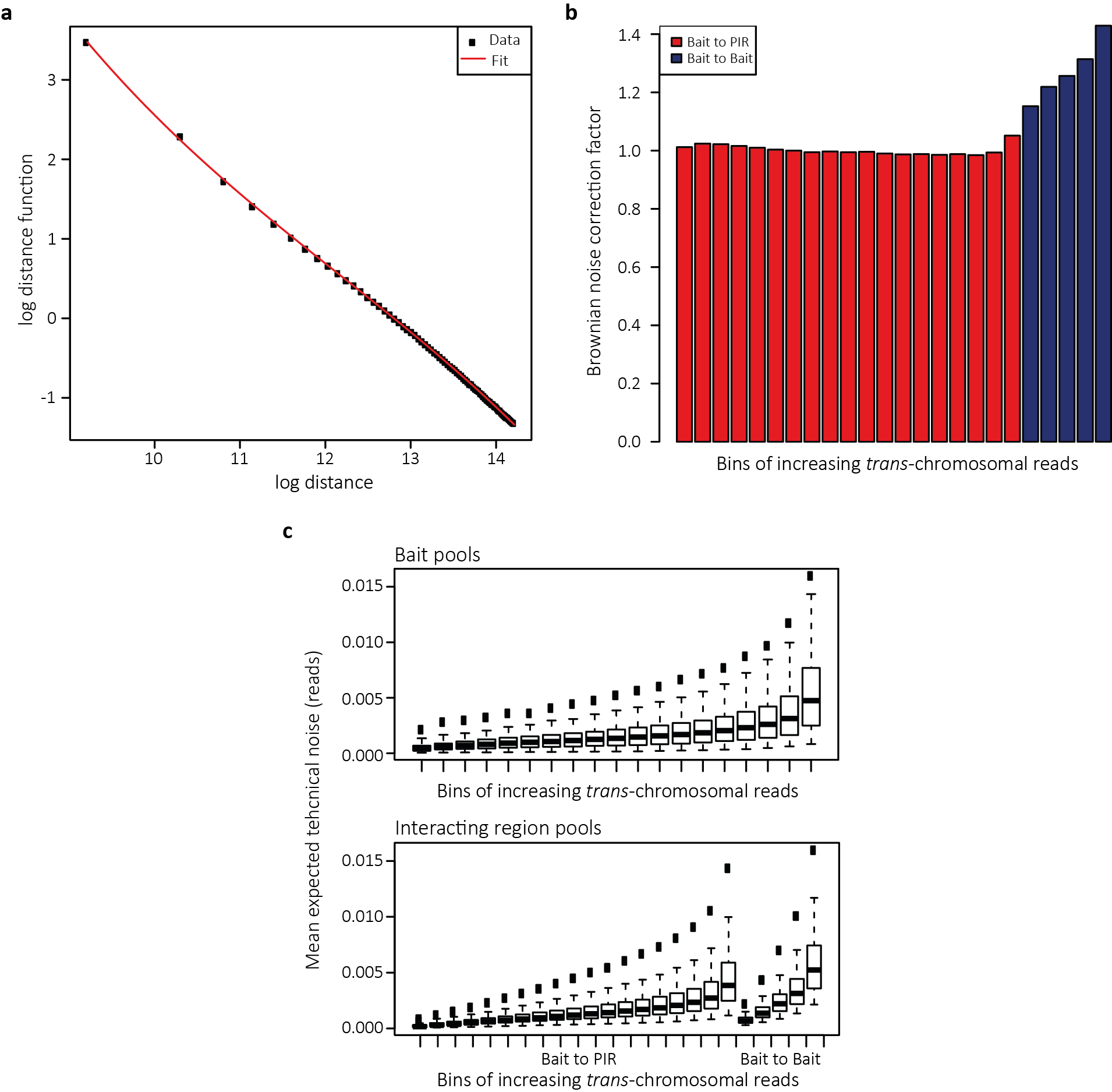

Supplement: Supplementary file 4 — Supporting information. [file BIT-118-784-s004.png]

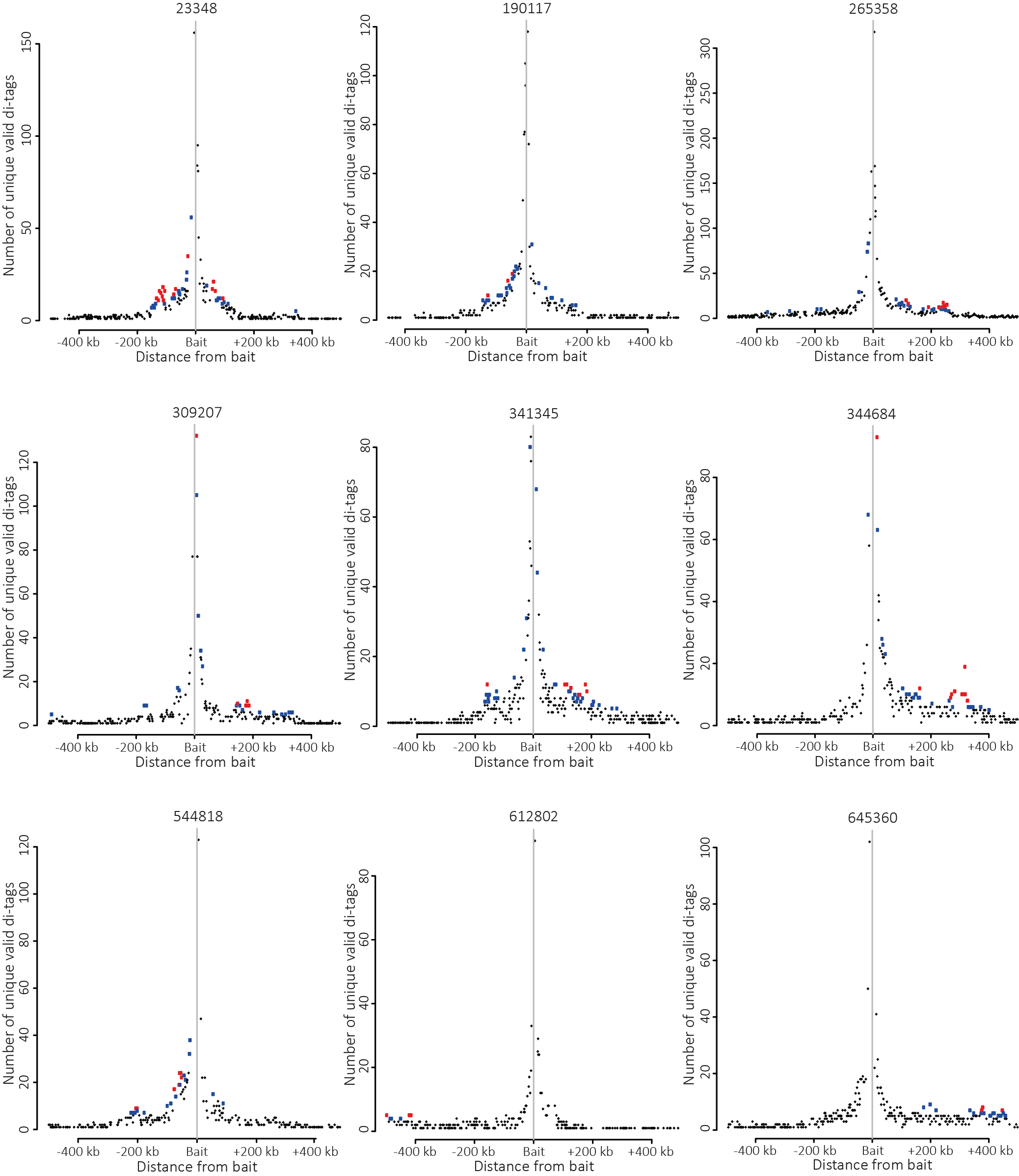

Supplement: Supplementary file 5 — Supporting information. [file BIT-118-784-s005.png]

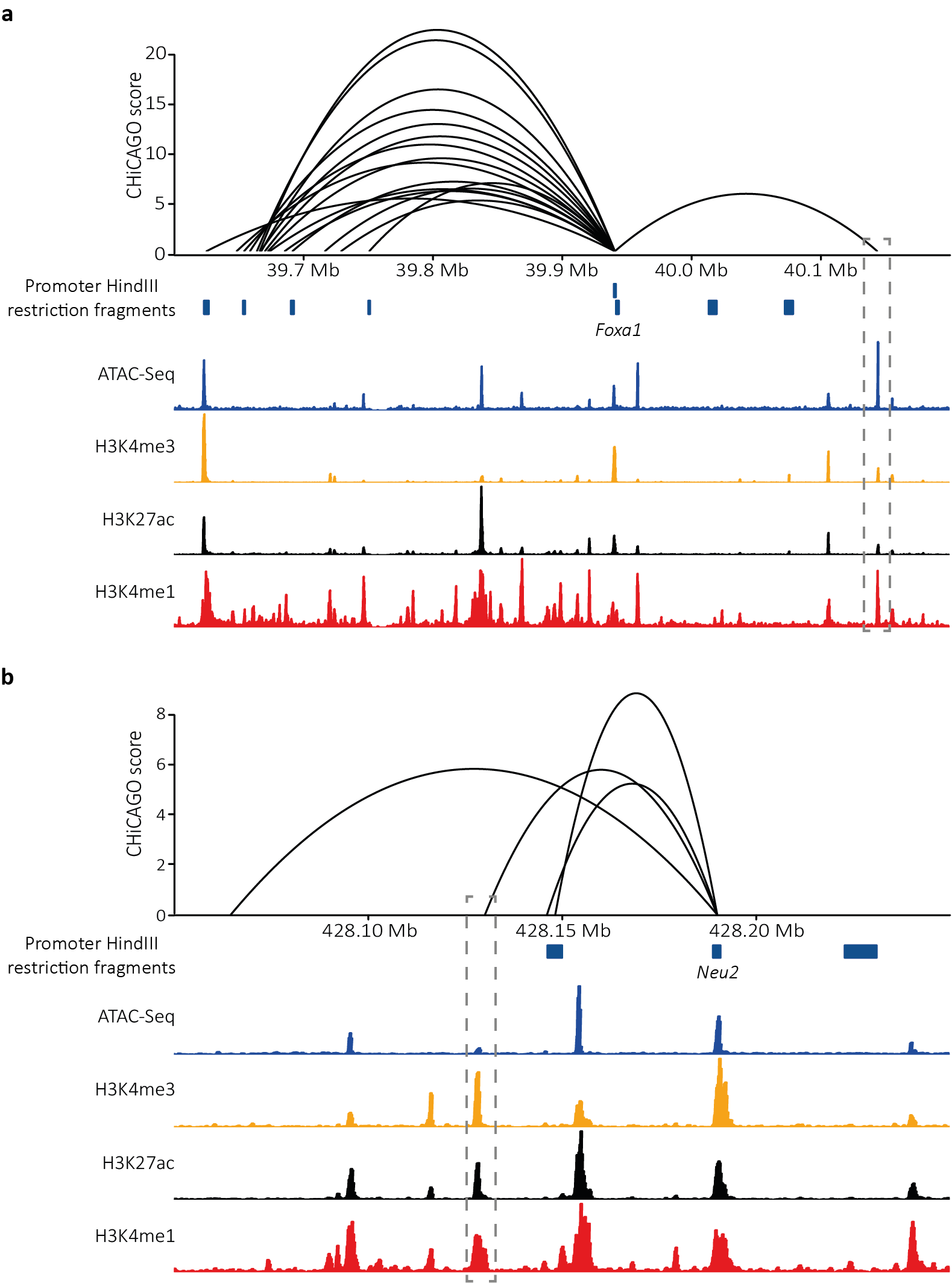

Supplement: Supplementary file 6 — Supporting information. [file BIT-118-784-s006.png]
